# Engineering a predatory bacterium as a proficient killer agent for intracellular bio-products recovery: The case of the polyhydroxyalkanoates

**DOI:** 10.1038/srep24381

**Published:** 2016-04-18

**Authors:** Virginia Martínez, Cristina Herencias, Edouard Jurkevitch, M. Auxiliadora Prieto

**Affiliations:** 1 Environmental Biology Department, Centro de Investigaciones Biológicas, CSIC, C/Ramiro de Maeztu 9, 28040 Madrid, Spain; 2 Department of Plant Pathology and Microbiology, Faculty of Agricultural, Food and Environmental Quality Sciences, the Hebrew University of Jerusalem, Rehovot 76100, Israel

## Abstract

This work examines the potential of the predatory bacterium *Bdellovibrio bacteriovorus* HD100, an obligate predator of other Gram-negative bacteria, as an external cell-lytic agent for recovering valuable intracellular bio-products produced by prey cultures. The bio-product targets to be recovered were polyhydroxyalkanoates (PHAs) produced naturally by *Pseudomonas putida* and *Cupriavidus necator*, or by recombinant *Escherichia coli* strains. *B. bacteriovorus* with a mutated PHA depolymerase gene to prevent the unwanted breakdown of the bio-product allowed the recovery of up to 80% of that accumulated by the prey bacteria, even at high biomass concentrations. This innovative downstream process highlights how *B. bacteriovorus* can be used as a novel, biological lytic agent for the inexpensive, industrial scale recovery of intracellular products from different Gram-negative prey cultures.

*Bdellovibrio bacteriovorus* is a small, highly motile, predatory bacterium that attacks other Gram-negative bacteria, invading their periplasm^1^,^2^. Once inside, the enzymatic degradation of prey constituents is initiated and the invader begins to grow, leading to the formation of a bdelloplast. The predator grows as a multinucleoid filament that finally septates to yield several progeny that escape the prey ghost to search for new prey cell (Fig. 1a–d). *B. bacteriovorus* was originally discovered in soil samples^3^ but has now been isolated from many environments, ranging from marine sediments to fresh water and even the guts of animals and humans^4^,^5^,^6^,^7^. This, together with an aptitude for preying on biofilms and multidrug-resistant pathogens, makes *B. bacteriovorus* a potential therapeutic agent for controlling human, animal and plant pathogens, the so-called “living antibiotic”^2^,^8^,^9^,^10^,^11^,^12^. In this work, we evaluated the potential use of a killer bacterium like *B. bacteriovorus* for biotechnological purposes. Given the predatory lifestyle of *Bdellovibrio* and its ability to lyse other bacteria, we investigated the feasibility for exploiting this predator as a novel downstream living lytic agent for the production of valuable intracellular bio-products (Fig. 2).

At the industrial scale, one of the most challenging downstream production processes is the isolation of bacterial polyesters or polyhydroxyalkanoates (PHAs). These biodegradable polymers, which are produced by Gram-negative and Gram-positive bacteria, are attractive alternatives to petroleum-based plastics^13^. They accumulate as intracellular granules in the bacterial cytoplasm and can account for up to 90% of cell dry weight. Different short-chain-length-PHAs (scl-PHA) such as poly-3-hydroxybutyrate (PHB) based bioplastics are currently produced at large scale by several companies (reviewed by^14^) and have extensive applications in packaging, moulding, fibre production and other commodities. Medium-chain-length-PHAs (mcl-PHA, with carbon numbers ranging from 6 to 14) are promising candidates as bioplastics given their longer-side-chain-derived properties of reduced crystallinity, elasticity, hydrophobicity, low oxygen permeability and biodegradability. They can be moulded and processed to make compostable packaging or resorbable materials for use in medical applications, and are already in use as food coatings, pressure-sensitive adhesives, paint binders and biodegradable rubbers^15^,^16^,^17^. Unconventional mcl-PHAs bearing bespoke functional moieties in their side chains can be produced using different biotechnological strategies^18^,^19^. However, their condition as intracellular bio-products makes their recovery difficult and expensive^20^,^21^. In the last decades, great effort has been made for the isolation of these biopolymers, which is the key step of the process profitability in the fermentation system^21^. Mechanical cell disruption by high-pressure homogenization is one of the most popular methods, although separation processes such us filtration, froth flotation, continuous centrifugation, enzymatic digestion or use of detergents and solvents have been also investigated^21^. Some disadvantages related to these systems are the high cost of the process or the severe reduction in polymer molecular weight. Several attempts have been carried out to mimic such procedures by using phage lysis genes to disrupt recombinant cells accumulating PHA^22^,^23^,^24^,^25^,^26^. However, these systems are species-specific and require engineering of the production chassis^26^, which limit the broad range applicability of this methodology. Here we present a robust and generalizable downstream system based on the use of the predatory bacterium *B. bacteriovorus* as a cell lytic agent. In contrast to phage-based methods, *Bdellovibrio* exhibits the advantage of being able to prey upon a wide range of Gram-negative bacteria^1^,^2^, which opens new avenues for the production and recovery of interesting compounds.

It has recently been shown that *B. bacteriovorus* HD100 can prey upon PHA-producers such as *Pseudomonas putida* KT2440 while the latter accumutales large amounts of mcl-PHA within its cells^27^. After lysing the prey, the predator hydrolyses and consumes some, but not all, of the PHA released into the extracellular environment; indeed, significant quantities of PHA granules and of free hydroxyalkanoic acid (HAs) oligomers (54% and 25% respectively of PHA accumulated by the prey bacteria)^27^ can be recovered. It has been suggested that this hydrolysis is the result of an extracellular-like mcl-PHA depolymerase (PhaZ_Bd_), which forms part of *B. bacteriovorus’s* hydrolytic arsenal^27^,^28^,^29^. The potential of this predator as a downstream tool for intracellular bio-product recovery is shown in the present work by engineering *B. bacteriovorus* HD100 to avoid it degrading the prey-produced PHA. We show the ability of *Bdellovibrio* to attack high cell density prey cultures, allowing the release of the polymer. To further demonstrate the feasibility of the system, engineered *Bdellovibrio* strains were also tested against other species of Gram-negative bacteria that accumulate these biopolymers. The results provide proof-of-principle that this system could be used in the production of PHA and other intracellular bio-products.

## Results

### Engineering *B. bacteriovorus* HD100 to prevent PHA hydrolysis during the predation of *P. putida* KT2440

**Figure 1:**
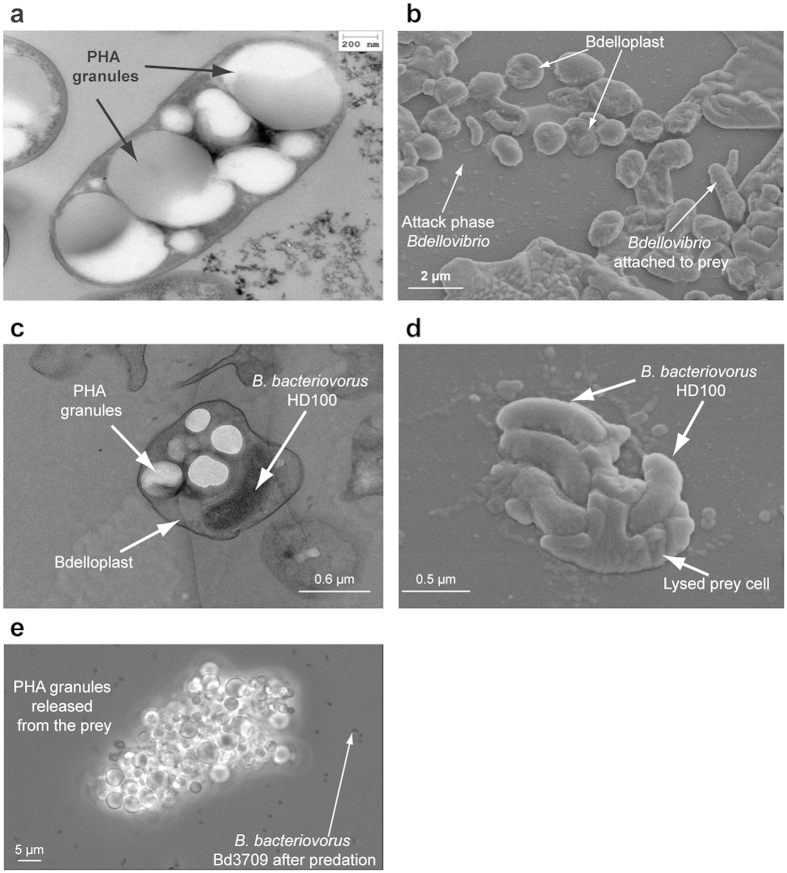
Different growth stages of *B. bacteriovorus* HD100 preying on *P. putida* KT2440. (**a**) TEM image of *P. putida* KT2440 accumulating mcl-PHA. (**b**) SEM image of a co-culture of *B. bacteriovorus* HD100 preying on *P. putida* KT2440. Different predator growth stages can be distinguished: Attack phase predator cells, entering the periplasm of the prey and growing in rounded prey cells (bdelloplast). (**c**) Detailed TEM image of predator cell development within a bdelloplast. (**d**) Detailed SEM image of prey cell lysis and release of predator progeny into the medium. (**e**) PHA granules released by *B. bacteriovorus* Bd3709 mutant after 24 h of predation upon *P. putida* KT2440.

*B. bacteriovorus* HD100 preying on PHA-accumulating *P. putida* cultures caused the release of part of the PHA granules and hydrolysis products (HAs, meaning a mixture of monomers and oligomers) into the culture medium (Table 1)^27^. To avoid the degradation of the prey-produced PHA by the attacking *B. bacteriovorus* cells, a *phaZ*_*Bd*_ depolymerase mutant strain - *B. bacteriovorus* Bd3709 - was produced by disrupting *phaZ*_*Bd*_ via the insertion of a kanamycin resistance gene (see Methods for details). After 24 h of predation, mutant *B. bacteriovorus* Bd3709 lysed the prey cells efficiently, resulting in a 2-logs increase in the predator cell number (Table 1). The *B. bacteriovorus* Bd3709/*P. putida* KT2440 co-culture showed PHA content similar to that of the control culture (*P. putida* without *B. bacteriovorus*) after 24 h of incubation [0.85 g l^−1^ (80% of the initial PHA produced by the *P. putida* KT2440 cells)] (Table 1). However, in *B. bacteriovorus* HD100/*P. putida* KT2440 co-cultures only 60% of the PHA content was recovered (Table 1). Figure 1e shows the remains of a *P. putida* KT2440 cell containing PHA after a being preyed by *B. bacteriovorus* Bd3709 mutant. Remarkably, PHA granules were visible in the extracellular medium together with the predator, while no integer prey cells were appreciable. A film showing *B. bacteriovorus* Bd3709 cells swimming around the released PHA granules can be seen in the Supplementary Material (Movie 2).

*B. bacteriovorus* HD100 released larger amounts of HAs into the extracellular medium than the Bd3709 strain, showing that *phaZ*_*Bd*_ disruption avoided PHA degradation during predation (Table 1). Indeed, with the Bd3709 mutant, nearly all the PHA granules accumulated by the prey were recovered after the predatory event. It is worth mentioning that *B. bacteriovorus* HD100 did not produce PHA, at least in the conditions assayed (see Methods for details).

### *B. bacteriovorus* HD100 can prey on high cell densities of *P. putida* KT2440 accumulating PHA

**Figure 3:**
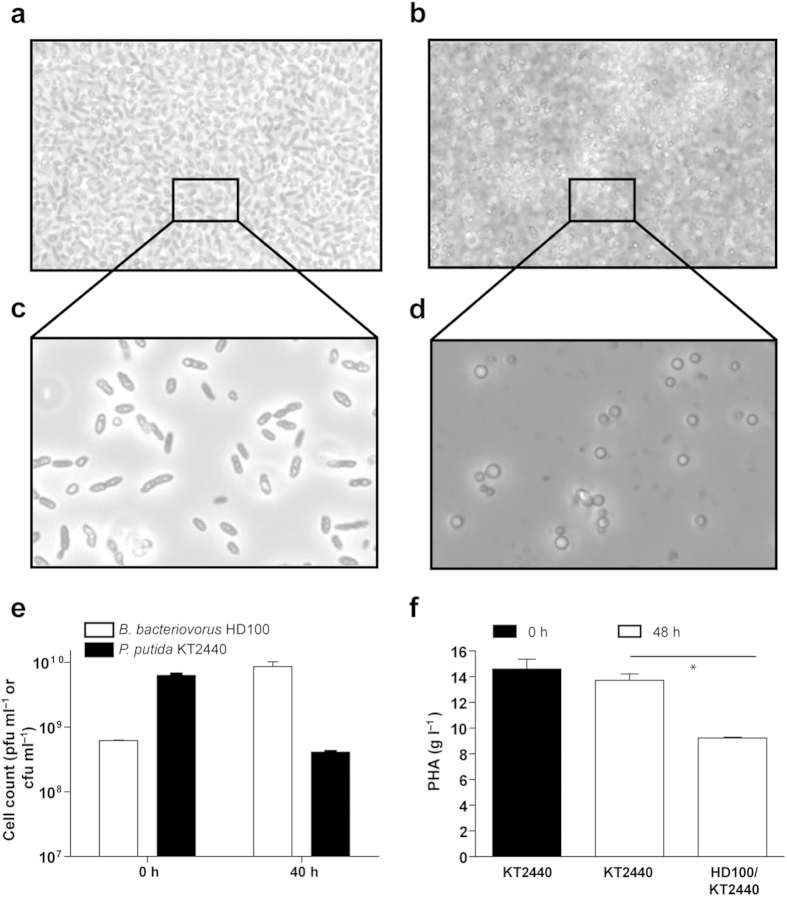
*B. bacteriovorus* HD100 preying on high cell densities of *P. putida* KT2440 accumulating mcl-PHA. (**a**) Phase-contrast microscopy of a co-culture of *B. bacteriovorus* HD100 preying on *P. putida* KT2440 at the onset of predation (time zero) and (**b**) after 40 h of incubation. (**c**,**d**) 1:100 dilution of the co-cultures from panels (**a,b**), respectively. Mcl-PHA granules can be observed in the extracellular medium after 40 h of predation. (**e**) Cell viability assay of the co-culture of *B. bacteriovorus* HD100/*P. putida* KT2440 (white bars and black bars, respectively) at the onset of predation (time zero) and within 40 h. (**f**) Total PHA content in the co-culture of *B. bacteriovorus* HD100/*P. putida* KT2440 compared to the control culture (KT2440 without the predator) at the onset of predation (time zero) (white bars) and within 40 h (black bars). Asterisks (^*^) indicate significant differences (^*^P < 0.05).

A requirement for the industrial scale use of *B. bacteriovorus* as a living, cell-lytic system would be the ability of the predator to attack high cell density prey cultures. Therefore, the potential of *B. bacteriovorus* HD100 to prey on high cell density cultures of *P. putida* KT2440 accumulating PHA was tested. For this, prey cultures were prepared in Hepes buffer with a cell biomass of 30.5 g l^−1^ (biomass production by PHA-producing pseudomonads under optimal conditions ranges between 15–55 g l^−1^)^30^, corresponding to 8.3 ± 0.3 · 10^9^ colony-forming units [(cfu) ml^−1^], with a PHA content of 15.1 g l^−1^ (Fig. 3a,c). These cultures were then inoculated with 6.3 ± 0.3 · 10^8^ plaque-forming units (pfu) ml^−1^ of *B. bacteriovorus* HD100. After 40 h of predation, a 1-log reduction in prey cells was observed while an increase of 1-log in the viable cell number of *B. bacteriovorus* was measured (Fig. 3e). This confirms the predator’s ability to prey on high-density cultures of PHA-accumulating *P. putida* KT2440. Examination of the co-cultures by phase-contrast microscopy clearly revealed the release of PHA granules into the extracellular medium (Fig. 3b,d). Although optimization of PHA recovery is required at this cell density scale, 65% of the PHA accumulated by the prey bacteria was recovered under our lab scale conditions (Fig. 3f). Notably, the polymer was directly extractable from the wet biomass of the co-cultures.

### Molecular characterization of the PHA recovered from predator/prey co-cultures

**Figure 4:**
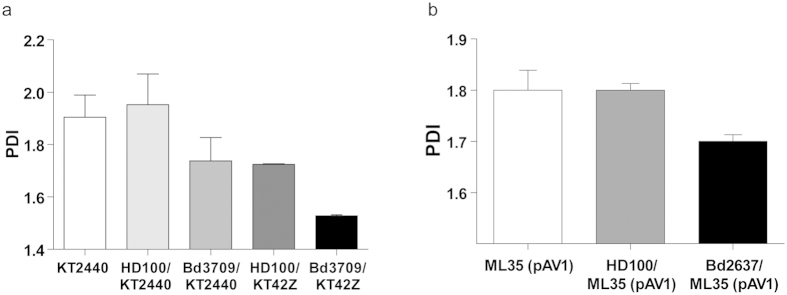
Molecular characterization of PHA and PHB polymers obtained from 24 h co-cultures with *B. bacteriovorus* strains. (**a**) Polydispersity index (PDI) values of the PHA obtained from the co-cultures of *B. bacteriovorus* HD100 and Bd3709 strains preying on PHA-accumulating *P. putida* KT2440 or KT42Z. (**b**) PDI values of the PHB obtained from *B. bacteriovorus* HD100 and Bd2637 strains preying on PHB-accumulating *E. coli* ML35 (pAV1). Polymer granules were directly isolated from the co-cultures sediments with chloroform (PHA) or dichloromethane (PHB). As an experimental control, the polymer was extracted from the prey culture (without predator) by breaking the cells with a French press. The results of one experiment are shown; the values were reproducible in three separate experiments with standard deviations of <10%. Error bars mean the variation of three technical replicates.

The PHA recovered from the *B. bacteriovorus* Bd3709/*P. putida* KT2440 co-culture was compared to that recovered from the *B. bacteriovorus* HD100/*P. putida* KT2440 co-culture and that from the *P. putida* KT2440 culture (as control experiment, extracted by subjecting the cells to lysis using a French press). The PHA extracted from *B. bacteriovorus* Bd3709/*P. putida* KT2440 co-culture showed a profile with a higher weight-average molecular weight (Mw) and number-average molecular weight (Mn), and a lower polydispersity index (PDI) than the PHA recovered from the *B. bacteriovorus* HD100/*P. putida* KT2440 co-culture (Fig. 4a and Supplementary Table 2). This indicates that, in the latter co-culture the biopolymer was partially degraded, increasing the heterogeneity of the polymer chains in terms of their molecular mass.

It is well documented that *P. putida* KT2440 possesses an intracellular mcl-PHA depolymerase (PhaZ_KT_) that plays a key role in PHA turnover^31^,^32^. PhaZ_KT_ degrades PHA and releases 3-hydroxycarboxylic acid monomers, which can either be oxidized via the β-oxidation pathway to generate energy, or be incorporated into nascent PHA polymer chains by PHA synthase, depending on the metabolic state of the cell^32^. To analyse the putative activity of PhaZ_KT_ depolymerase during the predatory event, the PDI of the polymer recovered from the co-cultures of *B. bacteriovorus* HD100 and Bd3709 preying on *P. putida* KT42Z (which lacks the PhaZ_KT_ depolymerase) was also quantified and characterized (Fig. 4a). As expected, the PHA extracted from the *B. bacteriovorus* Bd3709/*P. putida* KT42Z co-culture showed lower PDI than the PHA recovered from the *B. bacteriovorus* HD100/*P. putida* KT42Z co-culture, highlighting the extracellular-like depolymerase of *B. bacteriovorus* as the responsible for the partial degradation of the mcl-PHA during predation.

### Analysis of the mcl-PHA hydrolytic products recovered by *B. bacteriovorus* Bd3709

**Figure 5:**
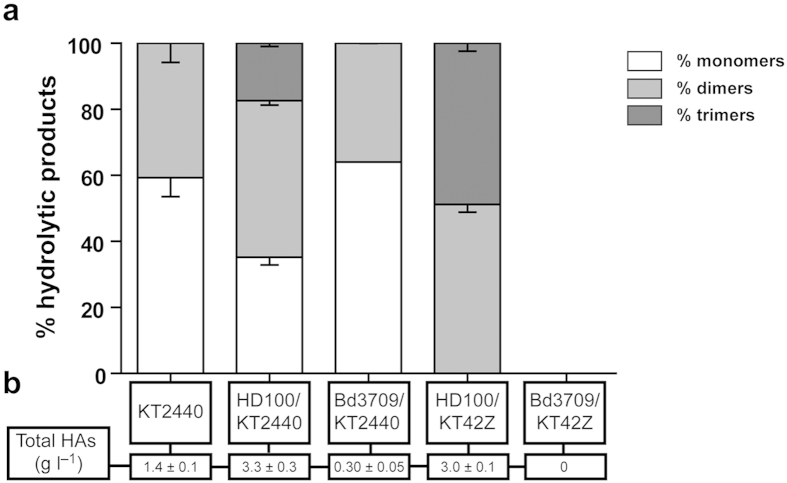
Mcl-PHA hydrolytic product profile identified in the co-culture supernatants of *B. bacteriovorus* strains preying on *P. putida* KT2440 and KT42Z accumulating mcl-PHA. (**a**) HPLC-MS analysis after 30 h of predation by *Bdellovibrio* strains. Monomers (white bars), dimers (light grey bars) and trimers (dark grey bars). Control supernatants of *P. putida* KT2440 and KT42Z are also shown. (**b**) Total PHA hydrolysis products quantified in the culture supernatants. No significant differences were observed between: (i) the percentage of monomers of the PHA extracted from KT2440 and Bd3709/KT2440, (ii) the percentage of dimers of KT2440 and HD100/KT2440, (iii) the percentage of dimers of KT2440 and Bd3709/KT2440, and (iv) the percentage of dimers of KT2440 and dimers of Bd3709/KT42Z. The rest of the conditions showed significant differences (P < 0.05) determined by ANOVA-test.

According to the above results, the profile of the HAs products should differ depending on the predatory strain used in the co-culture. To examine this, *P. putida* KT2440 cells were prepared in Hepes buffer at a biomass of 5.7 g l^−1^ (corresponding to 2.4 ± 0.2 · 10^7^ cfu ml^−1^ with a PHA content of 2.4 g l^−1^), and subsequently inoculated with 5.3 ± 0.3 · 10^7^ *B. bacteriovorus* HD100 or 3.7 ± 0.1 · 10^7^ pfu ml^−1^ *B. bacteriovorus* Bd3709. After 30 h of predation, the extracellular medium was analysed by HPLC-MS. The mcl-PHA hydrolytic product profile for the *B. bacteriovorus* Bd3709*/P. putida* KT2440 co-culture - mainly monomers and dimers - was similar to that recorded for *P. putida* KT2440 growing alone (Fig. 5). However, it differed strongly to that of the *B. bacteriovorus* HD100*/P. putida* KT2440 co-cultures, which showed larger proportions of dimers and trimers, similar to that obtained in *in vitro* experiments using pure PhaZ_Bd_ depolymerase^29^. These differences could be attributed to the different activities of the depolymerases produced by *P. putida* KT2440 (PhaZ_KT_, intracellular) and *B. bacteriovorus* (PhaZ_Bd_, extracellular-like) (see below).

The total PHA hydrolysis products released to the extracellular medium following predation was quantified by HPLC-MS (Fig. 5). Values of 3.3 ± 0.33 and 0.30 ± 0.05 g l^−1^ were recorded for the *B. bacteriovorus* HD100/*P. putida* KT2440 and *B. bacteriovorus* Bd3709/*P. putida* KT2440 co-cultures, respectively (Fig. 5b). To further confirm that PhaZ_Bd_ degrades PHA during predation, the PHA hydrolytic product profile was also examined for the co-cultures of *B. bacteriovorus* HD100 or Bd3709 preying on *P. putida* KT42Z (Fig. 5). As expected, no HAs were found in the *B. bacteriovorus* Bd3709/*P. putida* KT42Z co-culture, while the hydrolytic product profile of the *B. bacteriovorus* HD100/*P. putida* KT42Z co-culture showed (mainly) dimers and trimers (Fig. 5).

### Expanding the *B. bacteriovorus* toolbox to other microorganisms

Since *B. bacteriovorus* HD100 attacks a broad range of Gram-negative bacteria^1^,^2^, the feasibility of using this lytic tool in other production systems was tested. As a proof-of-concept, the recovery of other PHAs such as polyhydroxybutyrate (PHB), consisting of C4–C5 monomers and with different properties and applications^33^, was studied. *E. coli* ML35 (pAV1) expressing the three main proteins for PHB synthesis in *Cupriavidus necator* H16 (PhaC1, PhaA and PhaB1), was used as a prey bacterium. These cells were prepared in Hepes buffer at 5.25 g l^−1^ (corresponding to 2.2 · 10^9^ cfu ml^−1^ and 1.3 g l^−1^ PHB), and inoculated with 1.45 · 10^7^ pfu ml^−1^ of *B. bacteriovorus* HD100 for 24 h (Table 2). After 24 h of predation by *B. bacteriovorus* HD100 PHA content decreased by more than 50% of that previously accumulated by the prey, suggesting that the predator hydrolysed the PHB. In this case, the PHB hydrolysis observed cannot be ascribed to any hydrolytic activity of the prey, since *E. coli* strains are unable to degrade PHB due to the lack of the necessary depolymerases.

To investigate the effect of PHB degradation on the fitness of *B. bacteriovorus*, the wild-type strain *E. coli* ML35 (pACYC184) and the PHB-producing strain *E. coli* ML35 (pAV1), were cultured under PHB production growth conditions. Prey cultures were adjusted to equal residual (i.e., a PHB-free) biomass (0.3 ± 0.05 g l^−1^), and then inoculated with 1.9 ± 0.10^8^ pfu ml^−1^ of *B. bacteriovorus* HD100. After 24 h of predation, the predator population was 10-fold higher when preying on PHB-accumulating *E. coli* cells than on the wild-type *E. coli* cells unable to produce PHB (Supplementary Fig. 1). This suggests a benefit for *B. bacteriovorus* in terms of biomass yield when preying on bacteria containing an extra carbon source in the form of PHB. This differs to that reported with *P. putida*; when preying on this bacterium, the number of progeny is independent of the presence of mcl-PHA^27^.

A potential extracellular scl-PHA depolymerase coding sequence is found in the genome of *B. bacteriovorus* HD100 [open reading frame (ORF) Bd2637], adding to its hydrolytic arsenal^27^,^28^,^34^. This putative scl-PHA depolymerase might be responsible for the degradation of the prey’s PHB during the predatory cycle. Therefore, *B. bacteriovorus* was engineered to avoid PHB degradation via an in-frame deletion of ORF Bd2637. This was achieved by inserting the suicide vector pK18*mob*sacB-2637 into *B. bacteriovorus* HD100 via conjugation and homologous recombination, with subsequent sucrose suicide screening for gene replacement. After 24 h of predation upon *E. coli* ML35 (pAV1), *B. bacteriovorus* Bd2637 knockout caused the release of larger amounts of PHB than did *B. bacteriovorus* HD100 (64% and 48%, respectively) (Table 2). This shows that ORF Bd2637 codes for a genuine scl-PHA depolymerase and confirms *B. bacteriovorus* Bd2637 mutant can be used as an improved lytic agent in the PHB production process. These results demonstrate the potential of *B. bacteriovorus* as a tailored tool for use in the harvesting of prey intracellular products.

Predation in high-density PHB-producing *E. coli* ML35 (pAV1) cultures was also monitored. At the start of the experiment, host viability, cell biomass and PHB content were 2.6 · 10^9^ cfu ml^−1^, 55 g l^−1^ and 19 g l^−1^, respectively. After 48 h of predation, both the wild-type and mutant *B. bacteriovorus* strains lysed the prey cells efficiently, achieving 4-log reductions in prey cells.

The molecular characterization of the PHB recovered from the co-cultures of the wild-type and mutant *B. bacteriovorus* strains preying upon *E. coli* ML35 (pAV1) showed extremely high Mw, as described by reference^35^, ranging from 3 × 10^6^ to 1 × 10^6^, with relatively low PDI of around 1.8–1.7 (Fig. 4b and Supplementary Table 2). The PDI remained nearly unchanged during the predation event, independently of the predatory strain applied in the process. These results indicated that the impact of the functionality of the scl-PHA depolymerase in the molecular weight and PDI of the polymer is not significant in our assay conditions. This differs to the results obtained for *P. putida* (see above).

**Figure 6:**
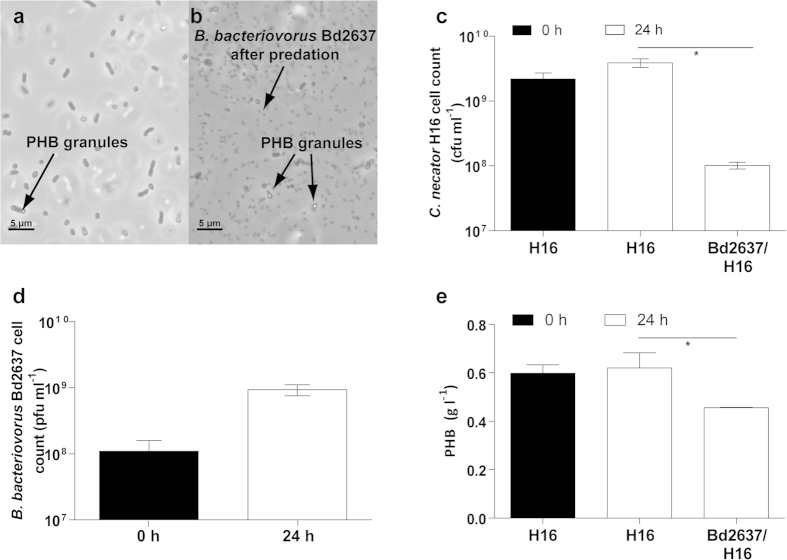
*B. bacteriovorus* Bd2637 preying on *C. necator* H16 accumulating PHB. (**a**) Phase-contrast microscopy of the co-culture at the onset of predation (time zero) and (**b)** after 24 h of incubation with *B. bacteriovorus*. (**c**) Number of viable cells of *C. necator* H16 accumulating PHB at time zero and after 24 h of predation without and with *B. bacteriovorus* Bd2637. (**d**) Number of viable cells of *B. bacteriovorus* Bd2637 at time zero and after 24 h of predation upon *C. necator* H16 accumulating PHB. (**e**) Total PHB content in the co-cultures. In all graphs, error bars indicate the standard deviation of the mean (n = 3). Asterisk indicate significant differences (*P ≤ 0.05) between control prey culture (i.e., without the predator) and *B. bacteriovorus* Bd2637 co-cultures, as determined by ANOVA-test.

Finally, other model PHB producer like *C. necator* H16 was grown under PHB production conditions and infected with *B. bacteriovorus* Bd2637. *C. necator* H16 (Fig. 6) felt prey to the predator demonstrating the versatility of this lytic system. Specifically, predation upon *C. necator* H16 let to an increase of 1-log in the viable cell number of *B. bacteriovorus* (Fig. 6d) and the recovery of 80% of the prey’s PHB after 24 h of predation (Fig. 6e). These results demonstrated the predator’s capability to prey upon other natural PHB producing bacteria, although scaling up would request to optimize the conditions of PHB production and recovery for each particular process.

## Discussion

The obligate predator *B. bacteriovorus* has long been proposed as an alternative for antibiotics, given its ability to attack Gram-negative bacteria^2^,^8^,^9^,^10^,^11^,^12^. The genome sequence of *B. bacteriovorus* HD100^28^ suggested that the vast amount of hydrolytic enzymes (biocatalysts) might be suitable for numerous industrial applications^27^,^28^,^29^. In this context, we highlight *B. bacteriovorus* as an innovative cell lytic agent for intracellular bio-products recovery. The system described in the present work consists of adding a predator culture to a culture of PHA-producing prey bacteria, leading to the release of this intracellular biopolymer into the culture medium. Using a *B. bacteriovorus* PhaZ_Bd_ depolymerase mutant (*B. bacteriovorus* Bd3709) to prevent the breakdown of the target product, more than 80% of the PHA accumulated in the prey cells was recovered from the extracellular medium. This system avoids the costly step of breaking the cells and drying the prey biomass – the conventional method of harvesting the PHA. Unlike *B. bacteriovorus* HD100, the Bd3709 mutant did not degrade the prey-produced mcl-PHA during predation, allowing the recovery of more homogeneous samples with lower PDI, a key requirement in polymer processing^21^.

In contrast to phage specificity, *B. bacteriovorus* attacks a wide range of Gram-negative bacteria^1^,^2^. Therefore, the cell lysis procedure was also successfully used in other prey species for the harvesting of PHB as *C. necator* H16. The system progressed successfully in the model strain *C. necator* H16 (Fig. 6). However, it is well known that during their cell cycle, some PHB producers might degrade the intracellularly accumulated PHB by the action of their native depolymerases^36^. This would make necessary to rational development of the phenotype of the producer prey or to engineer a more amenable heterologous chassis organism. The latter was attempted by expressing the PHB synthesis cluster from *C. necator* H16 in *E. coli* ML35 (*E. coli* is among the organisms most tractable to biological engineering^37^). Using this as the prey for *B. bacteriovorus* Bd2637 mutant (the HD100 knockout for the putative PHB depolymerase^29^,^34^), allowed the recovery of larger amounts of extremely high Mw (3–1 × 10^6^) PHB after 24 h of predation. However, this mutant still degraded some of the prey’s PHB. Indeed, *B. bacteriovorus* is known to have many hydrolases in its hydrolytic arsenal^28^, some of which might have degraded the PHB.

Unexpectedly, both *B. bacteriovorus* Bd3709 and Bd2637 killed more prey cells than did wild-type *B. bacteriovorus* HD100. Additional experiments are needed to determine whether mcl-PHA and scl-PHA depolymerase deletion improves the fitness and predation capacity of *Bdellovibrio*.

Previous studies of *B. bacteriovorus* HD100 preying upon *P. putida* showed that PHA degradation confers ecological advantages upon the former in terms of motility and predation efficiency, very likely due to an increment of the intracellular ATP content, but does not increase the biomass or number of predator cells^27^. In contrast, preying on PHB-accumulating *E. coli* cells did seem to afford the predator fitness benefits in terms of number of progeny, although the mean swimming speed was similar. The monomers and hydrolysis products derived from PHB degradation might be catabolised for biomass generation via a putative *R*-3-hydroxyacyl CoA synthase (Bd1803) [EC 6.2.1.3 (http://www.genome.jp/kegg-bin/show_pathway?bba00071)], followed by the action of a putative 3-hydroxyacyl-CoA isomerase to yield *S*-3-hydroxyacyl-CoA (Bd1836) [EC 5.1.2.3 (http://www.genome.jp/kegg-bin/show_pathway?bba00650)]. Finally, 3-hydroxyacyl-CoA dehydrogenase would convert *S*-3-hydroxybutyryl-CoA into acetoacetyl-CoA (Bd1836) [EC 1.1.1.35 (http://www.genome.jp/kegg-bin/show_pathway?bba00650)], which channels into central metabolism, thus explaining the increase in predator progeny after preying on PHB-accumulating *E. coli*. It has also been described that *B. bacteriovorus* uses intermediate metabolites it takes from the prey^38^,^39^; PHA and PHB may therefore have behaved as extra carbon sources. These metabolites might become incorporated into the fatty acid pathway and secondary metabolism, improving the predation capacity of the bacterium.

Although the examined system needs to be tested at larger scales in a pilot plant, it showed good performance at high biomass concentrations, suggesting industrial-scale upgrade is possible. This is worth investigating since it would provide a means of harvesting intracellular bio-products such as PHA in a single step, avoiding cell breakage and dry biomass treatments, and using reduced amounts of organic solvents. Not only would this be cheaper, it would have environmental benefits. More powerful predators also need to be developed, as do systems that can guarantee their long-term storage without loss of their predatory capacity. This opens new challenges to be addressed by synthetic biology strategies.

In summary, this study highlights the potential use of bacterial predators as external, living, cell-lytic agents of Gram-negative bacteria, causing the release into the extracellular medium of compounds previously made by the latter. The industrial-scale recovery of PHAs from bacterial cells is currently difficult and expensive. Solving this problem would unlock the economics of PHA production. The present work proposes an innovative downstream procedure that could make the production of bioplastics much profitable. This could have enormous environmental, economic and social benefits.

## Methods

### Bacterial strains, media and growth conditions

The bacterial strains and plasmids used in this work are described in Supplementary Table 1. Unless otherwise stated, *Escherichia coli*, *Pseudomonas putida* and *Cupriavidus necator* strains were grown in nutrient broth (NB) (Difco) or in lysogeny broth (LB)^40^ at 37 °C (*E. coli*) or 30 °C (*P. putida* and *C. necator*) with shaking. Chloramphenicol (Cm) (30 μg ml^−1^) and kanamycin (Km) (50 μg ml^−1^) were added when needed. Growth was monitored using a Shimadzu UV-260 spectrophotometer at 600 nm (OD_600_). Solid media were made with 1.5% (w/v) agar. For PHB production, *E. coli* ML35 (pAV1) was grown in LB broth with 1% glucose, at 20 °C for 40 h; *C. necator* was grown in a nitrogen-limited minimal medium modified from reference^41^ (0.33 g KH_2_PO_4_ l^−1^, 1.2 g Na_2_HPO_4_ l^−1^, 0.11 g NH_4_Cl l^−1^) for 24 h, supplemented with 3.25 mM MgSO_4_, a solution of trace elements (composition 1000 × 2.78 g FeSO_4_·7H_2_O l^−1^, 1.98 g MnCl_2_·4H_2_O l^−1^, 2.81 g CoSO_4_·7H_2_O l^−1^, 1.47 g CaCl_2_·2H_2_O l^−1^, 0.17 g CuCl_2_·2H_2_O l^−1^, 0.29 g ZnSO_4_·7H_2_O l^−1^) and fructose (55.5 mM) as the sole carbon source. For mcl-PHA production, *P. putida* strains were grown in 0.1 N M63, a nitrogen-limited minimal medium (13.6 g KH_2_PO_4_ l^−1^, 0.2 g (NH_4_)_2_SO_4_ l^−1^, 0.5 mg FeSO_4_·7H_2_O l^−1^, adjusted to pH 7.0 with KOH). This medium was supplemented with 1 mM MgSO_4_ and a solution of trace elements (composition 1000 × 2.78 g FeSO_4_·7H_2_O l^−1^, 1.98 g MnCl_2_·4H_2_O l^−1^, 2.81 g CoSO_4_·7H_2_O l^−1^, 1.47 g CaCl_2_·2H_2_O l^−1^, 0.17 g CuCl_2_·2H_2_O l^−1^, 0.29 g ZnSO_4_·7H_2_O l^−1^). Sodium octanoate (15 mM) was used as the sole carbon source, as previously described^27^,^42^. *B. bacteriovorus* HD100 was routinely grown in co-culture in Hepes buffer (25 mM Hepes amended with 2 mM CaCl_2_·2H_2_O and 3 mM MgCl_2_·3H_2_O, pH 7.8) or DNB liquid medium (consisting of 0.8 g l^−1^ NB supplemented with 2 mM CaCl_2_ and 3 mM MgCl_2_), with *P. putida* KT2440 as prey^27^. Prey cultures were prepared from cells grown in NB for 16 h, and diluted to OD_600_ 1 in Hepes buffer. After predation, the co-cultures were filtered twice through a 0.45 μm filter (Sartorius) and the *B. bacteriovorus* cells used in the assays below. All co-cultures were grown in 100 ml flasks in a final volume of 10 ml.

### DNA manipulations

DNA manipulations and other standard molecular biology techniques were essentially performed as previously described^40^. PCR amplifications were performed in the buffer recommended by the manufacturer plus 0.05 μg of template DNA, 2 U of AmpliTaq DNA polymerase and 0.5 μg of each deoxynucleotide triphosphate. Conditions for amplification were chosen according to the GC content of the oligonucleotides used. DNA fragments were purified by standard procedures using the Gene Clean Turbo Kit (MP Biomedicals). Genomic DNA was isolated with the Bacteria genomicPrep Mini Spin Kit (GE Healthcare). PCR products were purified using the High Pure Plasmid Isolation Kit (Roche Applied Science).

### Construction of *B. bacteriovorus* HD100 mutants

*B. bacteriovorus* Bd3709 (genotype *Bd3709*::pK18*mob*-3709) was constructed via the disruption of the *phaZ*_*Bd*_ gene (ORF Bd3709). This involved the insertion of the whole pK18*mob*-3709 plasmid (Supplementary Table 1) into the chromosome of the predator by conjugation and homologous recombination. The plasmid pK18*mob*-3709 carries a truncated version of the *phaZ*_*Bd*_ gene and its integration confers kanamycin resistance. The plasmid was constructed using the oligonucleotides PHO-F and PHO-R (Supplementary Table 1) with the *B. bacteriovorus* HD100 genome as a template. A 582 bp fragment of *phaZ*_*Bd*_ was produced by introducing an artificial stop codon to interrupt the translation of the gene in the resulting *B. bacteriovorus* Bd3709 mutant. This fragment was digested with the appropriate restriction enzymes and ligated using T4 DNA ligase, resulting in the desired mutant version of the gene. This was cloned into the corresponding *Hind*III and *Xba*I sites of the pK18*mob* plasmid to yield pK18*mob*-3709 (Supplementary Table 1) (the DNA sequence of the resultant plasmid was confirmed). This was then used to deliver the mutation to the host chromosome of *B. bacteriovorus* HD100. Biparental mating was performed using *E. coli* S17-λ*pir* as the donor strain and *B. bacteriovorus* HD100 as the recipient (Supplementary Table 1). For conjugation, 12 ml of the overnight culture of the donor strain, suspended in 100 μl of DNB, and 50 ml of *B. bacteriovorus* HD100/*P. putida* KT2440 co-culture filtered, pelleted and suspended in 100 μl of DNB, were collected on a Millipore filter, which was then placed on a DNB agar plate and incubated for 30 h at 30 °C. After incubation, the cells were suspended in 2 ml of DNB medium and plated on DNB plus kanamycin using the double-agar-overlay method^27^,^43^, with a kanamycin-resistant *P. putida* strain (from our laboratory collection) as the prey. Kanamycin-resistant conjugants were isolated from the double-layer agar plates and the insertion checked by PCR. To check the insertion stability, 10 consecutive passes from plate to plate containing DNB plus kanamycin were performed, and the insertion confirmed by PCR analysis of 20 mutant *B. bacteriovorus* plaques.

*B. bacteriovorus* Bd2637 (genotype Δ*Bd2637)* was constructed as follows. The *bd2637* gene coding for a putative scl-PHA depolymerase^29^,^34^ was inactivated by allelic exchange homologous recombination using the mobilizable suicide plasmid pK18*mob*sacB^44^. The deletion of ORF Bd2637 was engineered using the DNA fragments PHBA and PHBB (745 bp and 776 bp long respectively) generated by PCR using the primer pairs PHB-F and PHB-IR for PHBA, and PHB-IF and PHB-R for PHBB (Supplementary Table 1). These two fragments were digested with the appropriate restriction enzymes, ligated using T4 DNA ligase and cloned into the corresponding *Hind*III and *Xba*I sites of pK18*mob*sacB to yield pK18*mob*sacB-2637 (Supplementary Table 1). The resultant plasmid was confirmed by DNA sequencing and used to deliver the mutation to the host chromosome of *B. bacteriovorus* HD100 via homologous recombination. Biparental mating was performed for 30 h with *E. coli* S17-λ*pir* as the donor strain as described above. The strains resulting from the first recombination event were screened for the insertion of the knockout construct via PCR. Merodiploid clones were plated twice onto double-layer agar plates with a kanamycin-resistant *P. putida* strain as prey. Thereafter, the merodiploids were transferred onto double-layer agar plates containing 5% sucrose in the bottom layer, plus an additional 5% sucrose and *P. putida* cells in the top layer. The resulting turbid plaques were isolated and the second cross-over event confirmed by PCR, leaving the resultant mutant strain *B. bacteriovorus* Bd2637 with *bd2637* deleted (Supplementary Table 1).

### Predatory capability of *B. bacteriovorus* HD100 and mutant strains

*B. bacteriovorus* and prey strain viabilities in co-culture were calculated. Serial dilutions of the co-culture from 10^−1^ to 10^−7^ were made in DNB liquid medium. To calculate *B. bacteriovorus* viability, 0.1 ml of the appropriate dilution was mixed with additional 0.5 ml of prey cell suspension of *P. putida* KT2440 pre-grown in NB and prepared in Hepes buffer at OD_600_ 10, vortexed, and plated on DNB solid medium using the double-agar-overlay method. Predators were counted as pfu (plaque-forming units) developing on the lawn of *P. putida* KT2440 after 48 h of incubation at 30 °C. To calculate the viability of the prey strain, 10 μl of each dilution were placed on LB solid medium and the number of cfu (colony-forming units) counted. Experiments were performed in triplicate for each strain.

To test the predation capacity of the predator on prey cells containing PHA, *P. putida* and *E. coli* strains were grown under PHA production conditions, centrifuged, suspended in Hepes buffer (at different cell densities), and these cultured and inoculated with *B. bacteriovorus* strains (10^7^–10^9^ pfu ml^−1^). Interactions were left to proceed for 24–48 h. For predation experiments involving *C. necator* H16, the prey cultures were prepared in Hepes buffer (OD_600_ 1) and inoculated with 10^7^ pfu ml^−1^ of *B. bacteriovorus* HD100 for 24 h. To normalize the predation, results obtained with PHB-accumulating prey cells against those obtained with non-accumulating cells, *E. coli* ML35 (pACYC184) and *E. coli* ML35 (pAV1) prey suspensions were adjusted to have equal residual biomass (0.3 ± 0.05 g l^−1^) (i.e., the cell biomass after subtracting the mass of PHA) in Hepes buffer. These cultures were then exposed to 10^8^ pfu ml^−1^ of *B. bacteriovorus* strains for 24 h.

### Biomass calculation

Cell densities, expressed in grams of cell dry weight (CDW) per litre, were determined gravimetrically as previously reported^26^. Briefly, 10 ml of culture medium were centrifuged for 20 min at 14,000 *g* and 4 °C. The cell pellets and supernatants were lyophilised for 24 h and weighed. The residual biomass (total biomass minus the PHB biomass) of these cells was used in analyses of the capacity of *B. bacteriovorus* strains to prey on PHB-producing *E. coli* ML35 (pAV1).

### GC-MS analysis of PHA content

For total PHA quantification, cultures were harvested, lyophilized and analysed by gas chromatography-mass spectrometry (GC-MS) as previously reported^26^,^31^. For PHA quantification in *B. bacteriovorus* HD100 cells, 50 ml of a co-culture with *P. putida* KT2440 were filtered twice, lyophilized and analysed by gas chromatography-mass spectrometry.

### HPLC-MS analysis for the identification of PHA hydrolysis products

To identify degradation products, co-culture extracellular medium was analysed by high-performance liquid chromatography-mass spectrometry (HPLC-MS) as previously reported^27^,^29^. Briefly, the prepared extracellular medium was suspended in methanol at 3 mg/ml, and 25 μl injected into the chromatographic system. The separation of the hydrolysis products was performed using a Finnigan Surveyor pump coupled to a Finnigan LXQ TM ion trap mass spectrometer (Thermo Electron).

### PHA isolation and molecular characterization

10 ml of the co-cultures of *B. bacteriovorus* strains preying on *P. putida* KT2440 or *E. coli* ML35 (pAV1) accumulating PHA or PHB were centrifuged and the resulting sediments (wet biomass) suspended in 5 ml of chloroform or dichlorometane, respectively. The solvent phase was collected and precipitated by adding 10 volumes of methanol. Finally, the polymer was dried under vacuum for 10 min and analysed by GC-MS to determine the polymer content and purity. As a control, 10 ml of PHA-accumulating *P. putida* KT2440 or PHB accumulating *E. coli* ML35 (pAV1) cultures (without the predator bacterium) were harvested, suspended in salt solution, and disrupted twice by passing through a French pressure cell at 69 bars.

The weight-average molecular weight (Mw), number-average molecular weight (Mn) and polydispersity index (PDI) of the mcl-PHA were determined by size exclusion chromatography in a Perkin-Elmer instrument equipped with a series 200 isocratic pump connected to a differential refractometric detector (series 200a). Two Styragel columns (HR5E, 5 μm, 7.8 mm × 300 mm) (Waters) were conditioned at 70 °C and used to elute the samples (2 mg ml^−1^ concentration) at 0.7 ml min^−1^. HPLC grade N, N-dimethylformamide (DMF) supplemented with 0.1% v/v LiBr was used as the mobile phase. The calibration curve was constructed using polystyrene standards (Polymer Laboratories) from 580 to 1.6 · 10^6^ g mol^−1^. The sample injection volume was 100 μl. For PHB molecular characterization (Mw, Mn and PDI), the samples were analysed by employing gel permeation chromatography (GPC) coupled with a Waters HPLC 1512 equipped with a Waters 996 photodiode array detector and a TOSOH Bioscience TSKGEL GMHHR column (300 mm × 7.8 mm). Chloroform was used as elution solvent at a flow rate of 1 ml min^−1^. Briefly, samples were dissolved in chloroform at a final concentration of 0.5% (w/v) and filtered (PTFE membrane, 0.22 μm) prior to analysis. Calibration curves were performed with polystyrene standards at a range from 500 to 31.5 · 10^6^ g mol^−1^. Integration and molecular weight calculations were carried out using Empower GPC software (Waters).

### Phase contrast, scanning electron, and transmission electron microscopy

Cultures were routinely visualized using a 100X phase-contrast objective and images taken with a Leica DFC345 FX camera. For scanning electron microscopy (SEM), co-cultures of *B. bacteriovorus* HD100/*P. putida* KT2440 co-cultures were harvested, washed twice with distilled sterile water, fixed with 2% (w/v) paraformaldehyde for 2 h at room temperature, and washed three times with distilled sterile water. The dried samples were mounted on aluminium stumps and sputter-coated with chromium before SEM examination using a Philips XL 30 device (acceleration voltage 15 kV). For transmission electron microscopy (TEM), the same co-cultures were harvested, washed twice in PBS and fixed in 5% (w/v) glutaraldehyde in the same solution, as previously described^26^. The cells were incubated with 2.5% (w/v) OsO_4_ for 1 h, gradually dehydrated in ethanol solutions and propylene oxide, and embedded in Epon 812 resin. Ultrathin sections (50–70 nm) were cut and observed using a Jeol-1230 electron microscope.

### Statistical Analysis

Data sets were analysed using Prism 6 software (GraphPad Software Inc., USA). Comparisons between two groups were made using Student’s‐test. Comparisons between multiple groups were made using one‐way or two-way analysis of variance (ANOVA) test, depending whether one or two different variables were considered, respectively.

## Additional Information

**How to cite this article**: Martínez, V. *et al*. Engineering a predatory bacterium as a proficient killer agent for intracellular bio-products recovery: The case of the polyhydroxyalkanoates. *Sci. Rep*. **6**, 24381; doi: 10.1038/srep24381 (2016).

## Supplementary Material

Supplementary Information

Supplementary Movie S1

Supplementary Movie S2

## Figures and Tables

**Figure 2:**
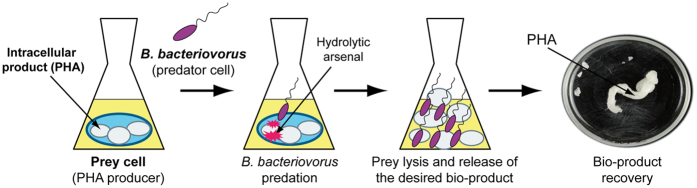
Illustration of the lytic system procedure based on the use of *B. bacteriovorus* for intracellular bio-products recovery. A culture of PHA-producing bacteria is prepared and infected with a suspension of *B. bacteriovorus* cells. After 24 h of predation the intracellular bio-product is released into the culture medium, facilitating the recovery.

**Table 1:** Growth and PHA content at time zero and 24 h of co-cultures involving *B. bacteriovorus* HD100 or the Bd3709 mutant preying on PHA-accumulating *P. putida* KT2440.

Strain cultures^a^	Prey biomass 0 h (g l^−1^)	Prey PHA content 0 h (g l^−1^)	PHA content in the bacterial sediment 24 h (g l^−1^)	PHA hydrolysis product contents in medium 24 h (g l^−1^)	Prey cell count 24 h (10^5^ cfu ml^−1^)	Predator cell count 24 h (10^8^ pfu ml^−1^)
KT2440^b^	1.92 ± 0.08	1.07 ± 0.11	0.87 ± 0.06	0.15 ± 0.090	1270 ± 423	–
HD100/KT2440	1.92 ± 0.08	1.07 ± 0.11	0.65 ± 0.05	0.38 ± 0.070	1.05 ± 0.84	26.20 ± 2.62
Bd3709/KT2440	1.92 ± 0.08	1.07 ± 0.11	0.85 ± 0.05	0.24 ± 0.020	0.09 ± 0.1	15.20 ± 4.11

^a^Viable prey cell numbers at time zero of predation: (9.5 ± 2.4) · 10^7^ cfu ml^−1^. Viable *B. bacteriovorus* HD100 cell numbers at time zero of predation: (8.3 ± 3.7) · 10^7^ pfu ml^−1^. Viable *B. bacteriovorus* Bd3709 cell number at time zero of predation: (4.4 ± 2.3) · 10^7^ pfu ml^−1^.

^b^Control prey cultures (i.e., with no predatory bacteria).

**Table 2:** Growth and PHB content at time zero and 24 h of co-cultures involving *B. bacteriovorus* HD100 or the Bd3709 mutant preying on PHB-accumulating *E. coli* ML35 (pAV1).

Strain cultures^a^	Prey biomass 0 h (g l^−1^)	Prey PHB content 0 h (g l^−1^)	PHB content in the bacterial sediment 24 h (g l^−1^)	Prey cell count 24 h (10^7^cfu ml^−1^)	Predator cell count 24 h (10^9^ pfu ml^−1^)
ML35 (pAV1)^b^	5.25 ± 0.13	1.3 ± 0.33	1.30 ± 0.22	283 ± 61	–
HD100/ML35 (pAV1)	5.25 ± 0.13	1.3 ± 0.33	0.63 ± 0.12	1.75 ± 0.645	13.2 ± 6.2
Bd2637/ML35 (pAV1)	5.25 ± 0.13	1.3 ± 0.33	0.83 ± 0.25	0.54 ± 0.51	16.1 ± 2.8

^a^Viable prey cell numbers at time zero of predation: (2.2 ± 4.0) · 10^9^ cfu ml^−1^. Viable *B. bacteriovorus* HD100 cell numbers at time zero of predation: (1.45 ± 3.4) · 10^7^ pfu ml^−1^. Viable *B. bacteriovorus* Bd3709 cell number at time zero of predation: (1.6 ± 3.3)·10^7^ pfu ml^−1^.

^b^Control prey cultures (without predator cells).
