# Author Correction: Engineering a predatory bacterium as a proficient killer agent for intracellular bio-products recovery: The case of the polyhydroxyalkanoates

**DOI:** 10.1038/s41598-022-06607-8

**Published:** 2022-02-10

**Authors:** Virginia Martínez, Cristina Herencias, Edouard Jurkevitch, M. Auxiliadora Prieto

**Affiliations:** 1 Environmental Biology Department, Centro de Investigaciones Biológicas, CSIC, C/Ramiro de Maeztu 9, 28040 Madrid, Spain; 2 Department of Plant Pathology and Microbiology, Faculty of Agricultural, Food and Environmental Quality Sciences, the Hebrew University of Jerusalem, 76100 Rehovot, Israel; 3 Present Address: Novo Nordisk Foundation Center for Biosustainability, Technical University of Denmark, Hørsholm, Denmark

Correction to: *Scientific Reports* 10.1038/srep24381, published online 18 April 2016

This Article contains errors.

During follow-up to the work described in this Article the Authors have sequenced the genomes of the strains derived and discovered that all of them (both the wild type and two mutant strains bd2637 and bd3709) derived from the parental predatory strain *Bdellovibrio bacteriovorus* 109J, and not HD100 as described in the Article.

Therefore, HD100 should be replaced with 109J everywhere in the Article apart from the instances outlined below.

In the final paragraph of the Introduction,

“It has recently been shown that *B. bacteriovorus* HD100 can prey upon PHA-producers such as *Pseudomonas putida* KT2440 while the latter accumutales large amounts of mcl-PHA within its cells^27^.”

should read:

“It has recently been shown that *B. bacteriovorus* can prey upon PHA-producers such as *Pseudomonas putida* KT2440 while the latter accumutales large amounts of mcl-PHA within its cells^27^.”

In the Results, under subheading ‘Expanding the B. bacteriovorus toolbox to other microorganisms’

“Since *B. bacteriovorus* HD100 attacks a broad range of Gram-negative bacteria^1,2^, the feasibility of using this lytic tool in other production systems was tested.”

should read:

“Since *B. bacteriovorus* attacks a broad range of Gram-negative bacteria^1,2^, the feasibility of using this lytic tool in other production systems was tested.”

In the same section,

“A potential extracellular scl-PHA depolymerase coding sequence is found in the genome of *B. bacteriovorus* HD100 [open reading frame (ORF) Bd2637], adding to its hydrolytic arsenal^27,28,34^.”

should read:

“A potential extracellular scl-PHA depolymerase coding sequence is found in the genome of *B. bacteriovorus* [open reading frame (ORF) Bd2637], adding to its hydrolytic arsenal^27,28,34^.”

Finally, in the Discussion,

“The genome sequence of *B. bacteriovorus* HD100^28^ suggested that the vast amount of hydrolytic enzymes (biocatalysts) might be suitable for numerous industrial applications^27–29^.”

should read:

“The genome sequence of *B. bacteriovorus*^28^ suggested that the vast amount of hydrolytic enzymes (biocatalysts) might be suitable for numerous industrial applications^27–29^.”

and

“Previous studies of *B. bacteriovorus* HD100 preying upon *P*. *putida* showed that PHA degradation confers ecological advantages upon the former in terms of motility and predation efficiency, very likely due to an increment of the intracellular ATP content, but does not increase the biomass or number of predator cells^27^.”

should read:

“Previous studies of *B. bacteriovorus* preying upon *P*. *putida* showed that PHA degradation confers ecological advantages upon the former in terms of motility and predation efficiency, very likely due to an increment of the intracellular ATP content, but does not increase the biomass or number of predator cells^27^.”

The sequencing data confirming the identity of the strains characterised in this Article can be accessed at Short Read Archive with the BioProject number PRJNA723206 (https://www.ncbi.nlm.nih.gov/sra/PRJNA723206).

These changes do not affect the conclusions of the Article.

