# Supplementary material for: Engineering a predatory bacterium as a proficient killer agent for intracellular bio-products recovery: The case of the polyhydroxyalkanoates
Source: Sci Rep. 2016 Apr 18;6:24381. doi: 10.1038/srep24381 (PMC4834554; doi:10.1038/srep24381)
Supplement: Supplementary Information [file srep24381-s1.pdf]

SUPPLEMENTARY INFORMATION FOR

Engineering a predatory bacterium as a proficient killer agent for intracellular bio-products recovery: The case of the polyhydroxyalkanoates

Virginia Martínez[1,3+], Cristina Herencias[1+], Edouard Jurkevitch[2] and M. Auxiliadora Prieto[1]*

[1]Environmental Biology Department, Centro de Investigaciones Biológicas, CSIC, C/ Ramiro de Maeztu 9, 28040 Madrid, Spain.[1]

[2]Department of Plant Pathology and Microbiology, Faculty of Agricultural, Food and Environmental Quality Sciences, the Hebrew University of Jerusalem, Rehovot 76100, Israel.

[3]Present address: V. Martínez, Novo Nordisk Foundation Center for Biosustainability, Technical University of Denmark, Hørsholm, Denmark.

*Corresponding author.

Mailing address: Department of Environmental Biology, Centro de Investigaciones Biológicas, CSIC, C/ Ramiro de Maeztu 9, 28040 Madrid, Spain.[1]

E-mail: auxi@cib.csic.es

Tel: +34 918 373 112; Fax: +34 915 360 432.

[+]Both authors contributed equally to this work.

Supplementary Table 1. Bacterial strains, plasmids and oligonucleotides used in this study.

**Strain, plasmid or oligonucleotide**	**Genotype, description or sequence (5' to 3')**	**Reference**
**Strain**		
B. bacteriovorus		
HD100	Type strain, genome sequenced	1,2
Bd3709	*Bd3709*::pK18*mob* mutant, HD100 derivative strain	This work
Bd2637	Δ*Bd2637* mutant, HD100 derivative strain	This work
***C. necator* H16**	Wild-type	3
E. coli		
ML35	*E. coli* B, *lacYI-*	Jurkevitch lab collection
S17-λ*pir*	*Tra*$^+$ *recA pro thi hsdR chr::RP4-2*	4
P. putida		
KT2440	*P. putida* mt-2 without TOL plasmid, *hsdR*	5
KT42Z	phaZ disruptional mutant, Km^r, KT2442 derivative strain	6
**Plasmid**		
pACYC184	p15A, Cm^R, Tet^R	8
pAV1	pACYC184 derivative containing PHB biosynthetic genes (*phbABC*) from *C. necator* H16, Cm^R	Prieto lab collection
pK18*mob*sacB	Km^R, ColE *ori*V, Mob+, *lac*Za, *sacB*; vector for allelic exchange homologous recombination mutagenesis	9
pK18*mob*sacB-2637	pK18*mob*sacB derivative used for Bd2637 deletion	This work
pK18*mob*	*ori*ColE1 Mob$^+$ *lacZα*$^+$, Km^R used for direct insertional disruption	9
pK18*mob*-3709	pK18*mob* derivative used for Bd3709 disruption	This work
**Oligonucleotide**[a]		
PHB-F	GCTCTAGAGACGTCCCAACCACCTCTTGAA	This work
PHB-IR	CGGGATCCGGTGACCTCCTGATGAAGAAATCA	This work
PHB-IF	CGGGATCCTGATTACGTCAGCAAATCCGG	This work
PHB-R	CCCAAGCTTGTTGTGAAACAGATTGCGCCC	This work
PHO-F	GCTCTAGAG GAGCTTCTTCCAGCCATTCGTGA CGAGC	This work
PHO-R	CCCAAGCTTCGGGATGACCCACGGTCATTC	This work

[a] Engineered endonuclease sites in the oligonucleotides are shown underlined.

Supplementary Table 2. Molecular characterization of mcl-PHA and PHB polymers.

Strain	Mn (kDa)	Mw (kDa)	PDI
KT2440 control	53.37 ± 7.54	100.37 ± 6.81	1.89 ± 0.13
HD100/KT2440	50.71 ± 4.48	96.41 ± 16.30	1.89 ± 0.16
Bd3709/KT2440	76.56 ± 11.30	124.80 ± 25.48	1.62 ± 0.15
HD100/KT42Z	82.57 ± 10.56	42.78 ± 10.32	1.73 ± 0.14
Bd3709/KT42Z	57.98 ± 6.59	88.57 ± 9.56	1.53 ± 0.13
ML35 (pAV1) control	2000 ± 300	3000 ± 200	1.8 ± 0.3
HD100/ML35 (pAV1)	600 ± 40	1060 ± 5	1.8 ± 0.1
Bd2637/ML35 (pAV1)	1000 ± 100	2000 ± 300	1.7 ± 0.1

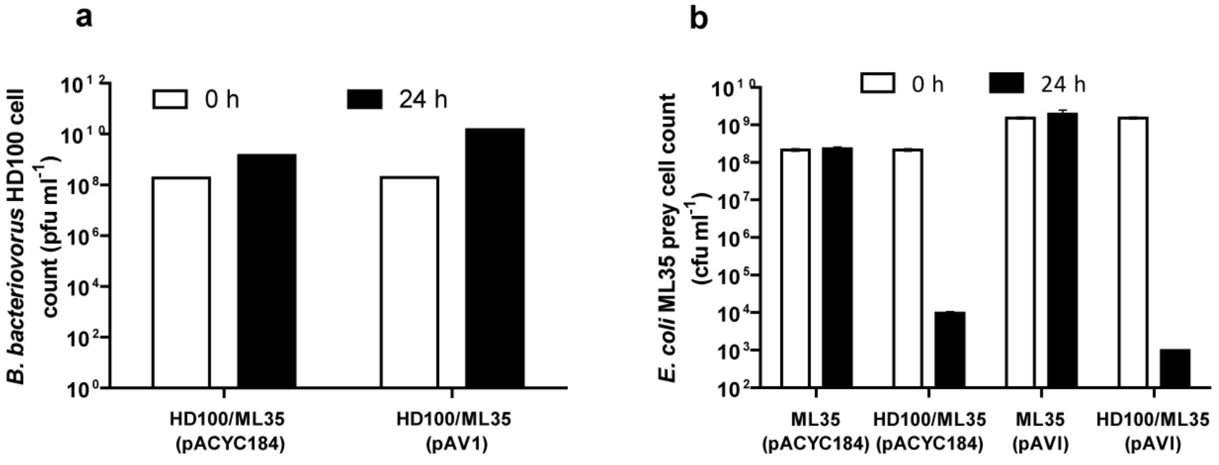

Supplementary Figure 1. Influence of prey PHB content on fitness of *B. bacteriovorus* HD100. (**a**) Number of viable cells of *B. bacteriovorus* HD100 at the start of the experiment (time zero) and after 24 h preying upon *E. coli* ML35 cells accumulating PHB [ML35 (pAV1)] or devoid of it [ML35 (pACYC184)], previously adjusted to 0.3 g l^{-1} of residual biomass. (**b**) Number of viable cells of *E. coli* ML35 accumulating PHB [ML35 (pAV1)] or devoid of it [ML35 (pACYC184)] at the start of the experiment (time zero) and after 24 h of predation with *B. bacteriovorus* HD100.

Supplementary movies:

Movie 1. *B. bacteriovorus* Bd3709 mutant preying on PHA-accumulating *P. putida* KT2440 at the onset of predation (time zero).

Movie 2. *B. bacteriovorus* Bd3709 mutant preying on PHA-accumulating *P. putida* KT2440 after 24 h of predation.

Supplementary references

1. Stolp, H. & Starr, M. P. *Bdellovibrio bacteriovorus* Gen. et Sp. n., a predatory, ectoparasitic, and bacteriolytic microorganism. *Antonie Van Leeuwenhoek* **29,** 217–248 (1963).
2. Rendulic, S. *et al*. A predator unmasked: life cycle of *Bdellovibrio bacteriovorus* from a genomic perspective. *Science* **303,** 689–692 (2004).
3. Poehlein, A., Kusian, B., Friedrich, B., Daniel, R. & Bowien, B. Complete genome sequence of the type strain *Cupriavidus necator* N-1. *J. Bacteriol.* **193,** 5017 (2011).
4. Simon, R., Priefer, U. & Pühler, A. A broad host range mobilization system for *in vivo* genetic engineering: transposon mutagenesis in gram-negative bacteria. *Nat. Biotechnol.* **1,** 784–791 (1983).
5. Bagdasarian, M. *et al*. Specific-purpose plasmid cloning vectors. II. Broad host range, high copy number, RSF1010-derived vectors for gene cloning in *Pseudomonas*. *Gene* **16,** 237–247 (1981).
6. de Eugenio, L.I. *et al*. Biochemical evidence that *phaZ* gene encodes a specific intracellular medium chain length polyhydroxyalkanoate depolymerase in *Pseudomonas putida* KT2442: characterization of a paradigmatic enzyme. *J. Biol. Chem.* **282,** 4951– 4962 (2007).
7. Chang, A. C. Y., & Cohen, S. N. Construction and characterization of amplifiable multicopy DNA cloning vehicles derived from the P15A cryptic miniplasmid. *J. Bacteriol.* **134,** 1141–1156 (1978).
8. Schäfer, A. *et al*. Small mobilizable multi-purpose cloning vectors derived from the *Escherichia coli* plasmids pK18 and pK19: selection of defined deletions in the chromosome of *Corynebacterium glutamicum*. *Gene* **145,** 69–73 (1994).
